# Role of Exosomes from Nucleus Pulposus Cells in Attenuating Intervertebral Disc Degeneration by Inhibiting Nucleus Pulposus Cell Apoptosis *via* the miR-8485/GSK-3β/Wnt/β-catenin Signaling Axis

**DOI:** 10.2174/0115665240370788250617070218

**Published:** 2025-06-23

**Authors:** Weiye Zhang, Ping Zhang, Jiawen Zhan, Xu Wei, Yuxuan Du, Ke Zhao, Liguo Zhu, Rong Xie, Hualong Xie, Shuaiqi Zhou, Gewen Wang, Chuhao Cai

**Affiliations:** 1 China Academy of Chinese Medical Sciences Wangjing Hospital, No. 6 Zhonghuan South Road, Chaoyang District, Beijing, China

**Keywords:** Nucleus pulposus cells, exosomes, intervertebral disc degeneration, traction force, miR-8485, Wnt/β-catenin signaling pathway

## Abstract

**Background:**

Studies have shown that abnormal stress is a significant inducer of Intervertebral Disc Degeneration (IVDD). Although traction force is commonly used to delay IVDD, its effects on Nucleus Pulposus Cells (NPCs) and their secreted exosomes remain unclear. In addition, this study systematically revealed the relationship between miR-8485 and IVDD for the first time.

**Methods:**

Cellular experiments were performed using a Flexcell cell stretching platform to apply traction force to NPCs. After optimizing loading parameters, NPC-derived exosomes (NPCs-exo) were isolated and subjected to miRNA high-throughput sequencing. Differentially expressed miRNAs were identified, and their regulatory effects on the Wnt/β-catenin pathway were investigated. *Ex vivo* rabbit spinal samples were used to validate the cellular experimental results under traction force loading.

**Results and Discussion:**

NPCs-exo were found to be internalized by NPCs, and traction force promoted NPCs-exo secretion. High-throughput sequencing and differential expression analysis identified miR-8485 as a differentially expressed miRNA in NPCs-exo secreted under Cyclic Mechanical Tension (CMT) conditions. Dual-luciferase reporter assays confirmed the targeted regulatory relationship between miR-8485 and GSK-3β, as well as its involvement in the Wnt/β-catenin pathway-mediated regulation of NPCs degeneration. *Ex vivo* experiments, including morphological and immunofluorescence analyses, revealed that the traction group exhibited better morphology than the pressure group, with a more organized AF, NP, and higher NPCs content, though some loss persisted. Both groups showed significant differences in ECM markers (Collagen II, Aggrecan, MMP3) compared to the control (*p* < 0.05). Additionally, the traction group had significantly higher Collagen II and Aggrecan levels than the pressure group (*p* < 0.05).

**Conclusion:**

CMT can promote the secretion of NPCs-exo, which are internalized by the NPCs. Through the delivery of miR-8485, NPCs-exo target and regulate GSK-3β, thereby enhancing Wnt/β-catenin pathway activity. This mechanism increases NPCs viability and extracellular matrix synthesis while suppressing apoptosis, ultimately delaying IVDD progression. Immunofluorescence staining in animal experiments confirmed that traction force effectively improves extracellular matrix expression in the IVD and mitigates stress-induced morphological alterations of the IVD.

## 1 INTRODUCTION

The Intervertebral Disc (IVD) is a crucial structure for bearing the body's load, with its development beginning in the third week of embryogenesis [1, 2]. It functions to absorb shock, distribute external forces, and maintain spinal stability [3, 4]. The Nucleus Pulposus (NP) is a gel-like avascular tissue located in the central region of the IVD. It is primarily composed of type II collagen, with small amounts of other collagen types and proteoglycans. Its main function is to distribute the body's load in various directions within each IVD under compressive forces [5]. As an avascular tissue, the NP relies on nutrient supply primarily through passive diffusion from the highly vascularized vertebral endplates via the Cartilaginous Endplate (CEP) located above and below it [6, 7].

Research indicates that abnormal stress is a significant inducer of Intervertebral Disc Degeneration (IVDD). A histological study on age-related changes in human IVDs demonstrated that reduced blood supply to the Cartilaginous Endplate (CEP) is a crucial factor leading to Nucleus Pulposus (NP) rupture during the second decade of life [8]. Our previous research also indicated that abnormal stress-induced degeneration of the CEP, which results in impaired nutrient metabolism and substance transport, plays a vital role in the onset and progression of IVDD [9]. Nucleus Pulposus Cells (NPCs), the primary cell type in NP tissue, are regulated by various factors within the complex microenvironment of the IVD [10, 11]. However, the detailed mechanisms underlying NPCs’ changes and degeneration under abnormal stress remain insufficiently understood. Investigating the effects of abnormal stress on the NPC microenvironment within the IVD can help elucidate the mechanisms of stress-induced IVD degeneration, laying a foundation for the prevention, treatment, and early repair of IVDD. Exosomes (EXO) are considered crucial mediators in local microenvironment regulation [12], and engineered EXO can deliver relevant drugs and small molecules to alleviate IVDD [13]. Studies have shown that human NPCs can secrete EXO, which can influence IVDD [14], and stress can regulate the secretion process of NPCs-exo, affecting the stability of the IVD microenvironment [15]. Current research suggests that EXO can influence IVDD by delivering miRNA. These miRNAs may play a pivotal role in modulating cellular processes within the disc, potentially offering a therapeutic avenue for mitigating the degenerative changes associated with IVDD [16-19].

As a form of abnormal stress, Cyclic Mechanical Tension (CMT) is involved in regulating the complex biological processes and signalling pathways of IVD [20]. However, its potential role in delaying IVDD progression remains unknown. Recent studies have shown that the activity and apoptosis of NPCs can be influenced by EXO from other cell sources [21], but the effects of NPCs-exo on NPCs under CMT conditions remain unclear. Following CMT loading, miR-8485 exhibited significant differential expression in NPCs-exo. Previous research indicates that miR-8485 can modulate the Wnt/β-catenin pathway, influencing chondrocyte function and arthritis progression. However, its potential involvement in IVDD regulation has not been investigated, leading to the initiation of this study. Therefore, this study aims to observe the changes in NPCs-exo under CMT stimulation and the effects of NPCs-exo on NPCs under co-culture conditions. By exploring the changes and interactions of NPCs and their autocrine EXO under CMT conditions, an *ex vivo* spinal unit loading and culture device (ESLCD) [22] was used to apply tensile loading to animal *ex vivo* spinal motion segments and observe the effects of tensile loading on the intervertebral discs and nucleus pulposus of animal spinal motion segments. Additionally, an animal IVDD model was prepared, and the effect of NPCs-exo injection on delaying IVDD in animals was observed.

## 2 MATERIALS AND METHODS

### 2.1 Cell Experiments

NPCs were isolated from traumatic lumbar disc surgeries performed at our hospital. Patients from whom NPCs were isolated underwent detailed examination and screening, following the methods outlined by Wan et al [23]. The degenerative condition of the IVD was assessed using Magnetic Resonance Imaging (MRI) according to the Pfirrmann grading system [24]. The inclusion criteria were as follows: 18 < age < 30, Pfirrmann grade = 1, and the NPCs were harvested from the L3-L5 intervertebral disc region. Informed consent was obtained from the patients, and the study protocol strictly adhered to the ethical requirements and was approved under the approval number WJEC-KT-2020-014-P001.

#### 2.1.1 Isolation and In vitro Culture of NPCs

Referring to published studies and methodologies [25], the excised NP tissue was meticulously cleared of any attached endplate and annulus fibrosus tissue under sterile conditions. The NP tissue was then finely minced with tissue scissors, washed in PBS, and transferred to centrifuge tubes containing type II collagenase to ensure thorough immersion in the solution. The tissue was digested at 37°C for 12 hours. The cell suspension was collected, and an equal volume of complete medium (10% fetal bovine serum + 100 U/mL penicillin-streptomycin + DMEM/F-12 medium) (Gibco, USA) was added. The mixture was centrifuged at 1200 r/minute for 6.5 minutes, and then 5 mL of complete medium was added to resuspend the cells. The cells were then seeded into 25 cm^2^ vented culture flasks and cultured at 37°C, 5% CO2, and 95% humidity. The culture medium was replaced every three days, and cell growth was monitored under a micro-scope. When NPCs reached 80%-90% confluency, they were plated onto Flexcell tension plates.

#### 2.1.2 CMT Loading of NPCs

The NPCs were seeded onto Flexcell tension plates at 50% confluency and incubated overnight at 37°C to allow for cell adhesion. Before applying CMT, the medium in the Flexcell tension cell culture plates (FTCCP) was replaced with 2 mL of corresponding serum-free medium to avoid interference from EXO present in the serum during subsequent NPCs-exo extraction. To determine the CMT parameters that have minimal impact on NPCs’ activity, the strain rate and frequency parameters were screened in two steps. The frequency was set to 1 Hz. The strain rate parameter in Step 2, denoted as X, was determined based on the screening results from Step 1. The determination of the strain rate depends on NPCs' cell viability, as assessed by the CCK-8 assay, to select the optimal strain rate parameter. To eliminate any potential impact of the Flexcell tension plates on cell activity, a control group was established (Table **1**).

#### 2.1.3 Measurement of Proliferation Activity of NPCs

After digestion with trypsin, NPCs loaded with CMT were seeded at 5×10^3^ cells per well in sterile 96-well cell culture plates and incubated for 12 hours. Subsequently, each well was supplemented with serum-free DMEM medium containing 10 µL of CCK-8 reagent, followed by further incubation at 37°C in the dark for 2 hours. Absorbance was measured at 450 nm using a microplate reader.

#### 2.1.4 Isolation of NPCs-exo

Using the EXO Extraction Kit (China Meilun Biotech), NPCs-exo were separated and purified using magnetic bead-based isolation. Supernatants of NPCs cultures post-loading were collected using a pipette, 550 µL of which were transferred to a new 1.5 mL centrifuge tube and centrifuged at 4°C, 3000 grams for 10 minutes. The resulting 500 µL supernatant was then transferred to a new 1.5 mL centrifuge tube for further processing. Following the protocol of the EXO Extraction Kit, NPCs-exo were separated into CMT-NPCs-exo and NPCs-exo groups in the centrifuge tubes containing pre-treated samples.

#### 2.1.5 Characterization and Identification of NPCs-exo

50 µL of NPCs-exo suspension was dropped onto a copper grid, allowed to stand at room temperature for 1 minute, and the excess liquid was removed from the side using filter paper. Approximately 20 µL of 2% phosphotungstic acid solution (pH 6.8) was applied to the grid and stained negatively for 1 minute at room temperature. The excess stain was blotted with filter paper, and the grid was allowed to air dry. Furthermore, it was observed and photographed under a transmission electron microscope (HT7830, Hitachi, Tokyo, Japan). All protein expression levels were determined via Western blot analysis. The total protein of cells and exosomes was extracted using the BCA protein extraction kit (Beyotime Biotechnology, China). The protein samples were added to the loading buffer and boiled for 5 minutes. Following the treatment, the proteins were separated using sodium dodecyl sulfate-polyacrylamide (SDS) gels (12%) by polyacrylamide gel electrophoresis (PAGE, Beyotime Biotechnology, China). The proteins were transferred to polyvinylidene fluoride membranes (PVDF membranes, Millipore, United States) using a wet blotting method. Following blocking with 5% non-fat milk in Tris-buffered saline containing 0.1% Tween-20 (TBST) for 1 hour, the membranes were incubated overnight at 4°C with suitable primary antibodies. After being washed 3 times in TBST, the membranes were incubated with the secondary antibodies for 2 hours at room temperature. After adding ECL luminescence solution, the bands were detected using the Li-Cor Odyssey 9120 Infrared Imaging System (Bio-Rad, United States). The intensity of bands was quantified through the ImagePro Plus software (Version 6.0, Media Cybernetics, United States). The antibodies used are as follows: Calnexin (1:1,000, Abcam, United Kingdom), CD63 (1:1,000, Abcam, United Kingdom), and TSG101 (1:1,000, Abcam, United Kingdom). The antibody for GAPDH (1:2000; Abcam, United Kingdom) was used as a control.

#### 2.1.6 Flow Cytometry Detection

200 μL of the homogenized NPCs-exo suspension was taken per tube after separation and purification. 0.2 μL of CFSE staining reagent (Solarbio) was added to each tube, which were then incubated at 37°C in the dark for 10 minutes, followed by detection using a flow cytometer. The CMT group, the No-CMT group, and the blank group were set up. For the blank group, neither dye nor the test sample was added. Flow cytometric detection was then performed.

#### 2.1.7 NPCs-exo Size Detection

The diluted NPCs-exo solution was introduced into a Nanoparticle Tracking Analysis (NTA) instrument to detect the size of the exosomes. The working principle of NTA involves collecting the scattered light signals from the particles, observing the Brownian motion of the exosomes in the solution, and performing tracking and analysis.

#### 2.1.8 PKH67 Fluorescent Labelling of NPCs-exo

NPCs-exo were labelled according to the instructions of the PKH67 fluorescent dye kit (Fluorescence). Specifically, the PKH67 reagent was allowed to reach room temperature, then diluted 10-fold with the diluent provided. Next, the PKH67 stock solution was diluted 25-fold with PBS to prepare the staining working solution. The prepared NPCs-exo were resuspended in 100 μL of the staining working solution and incubated at 37°C for 5 minutes, followed by incubation at 4°C for 15 minutes. After staining, 400 μL of PBS was added.

#### 2.1.9 Observation of NPCs Uptake of NPCs-exo

NPCs were cultured following method 3.1. When the cell density reached 80%, the cells were passaged at a 1:3 ratio. PKH67-labelled and purified NPCs-exo were used. 500 μL of NPCs-exo was added to NPCs at 30% cell density and co-cultured for 24 hours. The following experimental groups were set up, following dissociation of the cells with trypsin into a single cell suspension using the culture medium. The cell suspension was smeared directly onto slides, and fluorescence imaging was performed under an inverted fluorescence microscope. The NPCs co-cultured in each group were analyzed for apoptosis rate and cell proliferation activity using a flow cytometer.

#### 2.1.10 qPCR Detection

Total RNA was extracted using the TRIzol method (Takara, Japan) according to the manufacturer's instructions. cDNA synthesis was performed using a reverse transcription kit (Promega, USA). The reaction mixture was then placed in a quantitative real-time PCR (qPCR) instrument. PCR cycling conditions were set as follows: initial denaturation at 95°C for 10 minutes, followed by 45 cycles of denaturation at 95°C for 15 seconds, and annealing/extension at 60°C for 1 minute. qPCR primer sequences were synthesized by Shanghai Sangon Biotech Co., Ltd. Untreated NPCs served as both the blank control and positive control groups. GAPDH was used as the internal reference gene. The relative mRNA expression levels of Aggrecan, Collagen II, and MMP3 in NPCs from each experimental group were calculated using the 2^(-ΔΔCt) method (Table **2**).

#### 2.1.11 High-throughput Sequencing and Analysis of NPCs-exo miRNA

Total RNA was isolated from NPCs-exo using the Total EVs RNA Isolation Kit (RiboBio, China). RiboBio assessed the quality and quantity of the miRNA and constructed and sequenced the miRNA library. The library was sequenced using the HiSeq 2500 system (Illumina, USA), and the Illumina analysis software was applied to the raw read data. Data normalization: The miRNA sequencing data were normalized using the “limma” package in R language. Differential expression analysis: The DESeq2 software was used for differential analysis of miRNA between the two groups, with screening criteria of |log2(Foldchange)| > 1 and p < 0.05. Potential target genes of the differentially expressed miRNAs were predicted using databases such as TargetScan, miRDB, miRbase, miRTarBase, miRWalk, and miRPathDB. Gene Ontology (GO) analysis was performed to identify the potential functions of the target genes. The Kyoto Encyclopedia of Genes and Genomes (KEGG) was used to predict the signalling pathways involved with the target genes.

#### 2.1.12 Dual-luciferase Reporter Assay

Wild-Type (WT) or Mutant (MT) GSK-3β transfection plasmids were constructed. The miRNA mimics and GSK-3β overexpression plasmid were synthesized by iScienceX (Beijing) Co., Ltd. NPCs were seeded in a 6-well plate 24 hours before transfection. When the cells reached 40-50% confluency, the miRNA mimics were transfected according to the manufacturer's instructions using Lipofectamine 2000 (Invitrogen, USA). The cells were harvested 48 hours after transfection for subsequent experiments.

### 2.2 Animal Experiments

#### 2.2.1 Experimental Animals and Tissue Samples

All experimental procedures were conducted in accordance with the institutional guidelines for the care and use of laboratory animals at the China Academy of Chinese Medical Sciences. The experimental subjects consisted of 27 male New Zealand White rabbits, each approximately 18 weeks old and weighing around 3.0 kg. The utilization of these animals followed the protocol approved by the Animal Ethics Committee of China Academy of Chinese Medical Sciences (No: 20180406). Prior to the experiment, the rabbits were housed for one week in an environment where the temperature was maintained between 18-24°C and the relative humidity ranged from 40-70%. The facility provided adequate ventilation and a 12-hour light/12-hour dark cycle. Each rabbit was caged individually to ensure ample space for movement, as well as ease of cleaning and disinfection. The rabbits were allowed access to food and water.

The animals were anesthetized with pentobarbital (100 mg/kg). Five minutes prior to euthanasia, 25,000 IU of heparin was administered via the auricular vein. Following euthanasia by CO_2_ asphyxiation, the soft tissues and posterior elements of the spine were excised. In accordance with previously published sampling methods [26, 27], a single motion segment (L4/5) was harvested from each rabbit for analysis. These segments encompassed the entire intervertebral disc, including the CEP, AF, and NP, as well as the adjacent vertebral bodies. As it was an *in vitro* experiment, all the rabbits were euthanized, and the spinal column samples were systematically dissected and collected, and then randomly assigned to different groups for experimentation. During the experimental process, a blind design was implemented, where the individual responsible for grouping and the one conducting the experiments were kept separate, ensuring that the experimenter remained unaware of the group assignments.

#### 2.2.2 Preparation of Ex Vivo Segment Culture Medium

Based on the established protocol [28], a culture medium suitable for the specimens was prepared. Dulbecco's Modified Eagle's Medium (DMEM) was supplemented with 50 mg/mL L-ascorbate, 100 U/mL penicillin, 100 mg/mL streptomycin, and 2.5 mg/mL Fungizone. The osmolarity of the DMEM was adjusted to 410 mOsm/kg through the addition of NaCl.

#### 2.2.3 Ex Vivo Spinal Loading Device for Rabbit Spinal Motion Segments (ESLCD)

The ESLCD, an upgrade of a previously published design [9], features fully automated mechanical loading and culture capabilities under sterile conditions (Patent No. CN115372149B). Following thorough cleaning and high-temperature sterilization of all ESLCD components, the FSUs were secured to the culture bottles of the ESLCD using dental model resin (Type II) in a laminar flow hood. The culture bottles were then properly positioned in the ESLCD and connected to the culture medium circulation system. To simulate the *in vivo* FSU environment, a continuous 300N compressive load was applied to all groups except the pressure group, following our previously published protocols [28]. Based on our previous research findings [29], the traction force parameter was set to 10 kg (100 N), which is considered appropriate for the IVD traction force in animal models. This level of force provides effective biomechanical stimulation without causing tissue damage. The traction force group received an additional 10 kg traction force for 30 minutes daily, in addition to the baseline compression. The culture medium was replaced daily, and the culture environment was maintained at 37°C with 5% CO_2_ in an incubator. The control group underwent the same medium replacement frequency and loading environment as the pressure and traction force groups. Samples were collected for analysis on days 0 and 7 of loading.

#### 2.2.4 Histomorphological Analysis

At each time point, motion segments were extracted from each group (n = 4) and fixed overnight in 10% formalin. Decalcification was performed in 19% EDTA for up to 21 days, followed by paraffin embedding. Mid-sagittal sections were cut at 4 μm intervals and subjected to Hematoxylin and Eosin (H&E) staining and Safranin O-Fast Green staining.

#### 2.2.5 Immunofluorescence Staining

At each time point, motion segments were extracted from each group (n = 4). Standard procedures were employed for deparaffinization and rehydration. Antigen retrieval was performed using EDTA antigen retrieval buffer (pH 9.0) under high-pressure conditions. Immunofluorescence staining was then performed. Images were captured using an inverted fluorescence microscope (Olympus Corporation, Japan) with the following excitation and emission wavelengths: UV excitation 330-380 nm, emission 420 nm; FITC green light excitation 465-495 nm, emission 515-555 nm; CY3 red light excitation 510-560 nm, emission 590 nm. For each fluorescence image, three random equal areas were selected for each indicator. Image-Pro Plus software was used to analyze the images and record the mean optical density for each indicator across different groups. Statistical analysis was subsequently performed on these data.

### 2.3 Statistical Analysis

Statistical analysis was performed using SPSS software, version 22.0 (IBM Corp., Armonk, NY, USA). Data collected from a minimum of three independent experiments were expressed as mean ± standard deviation (SD). For comparisons between two groups, Student’s t-test was utilized, while Analysis of Variance (ANOVA) was applied for comparisons among multiple groups. A P-value of less than 0.05 was considered statistically significant.

## 3 RESULTS

### 3.1 Isolation and Characterization of NPCs-exo

**Fig. (1):**
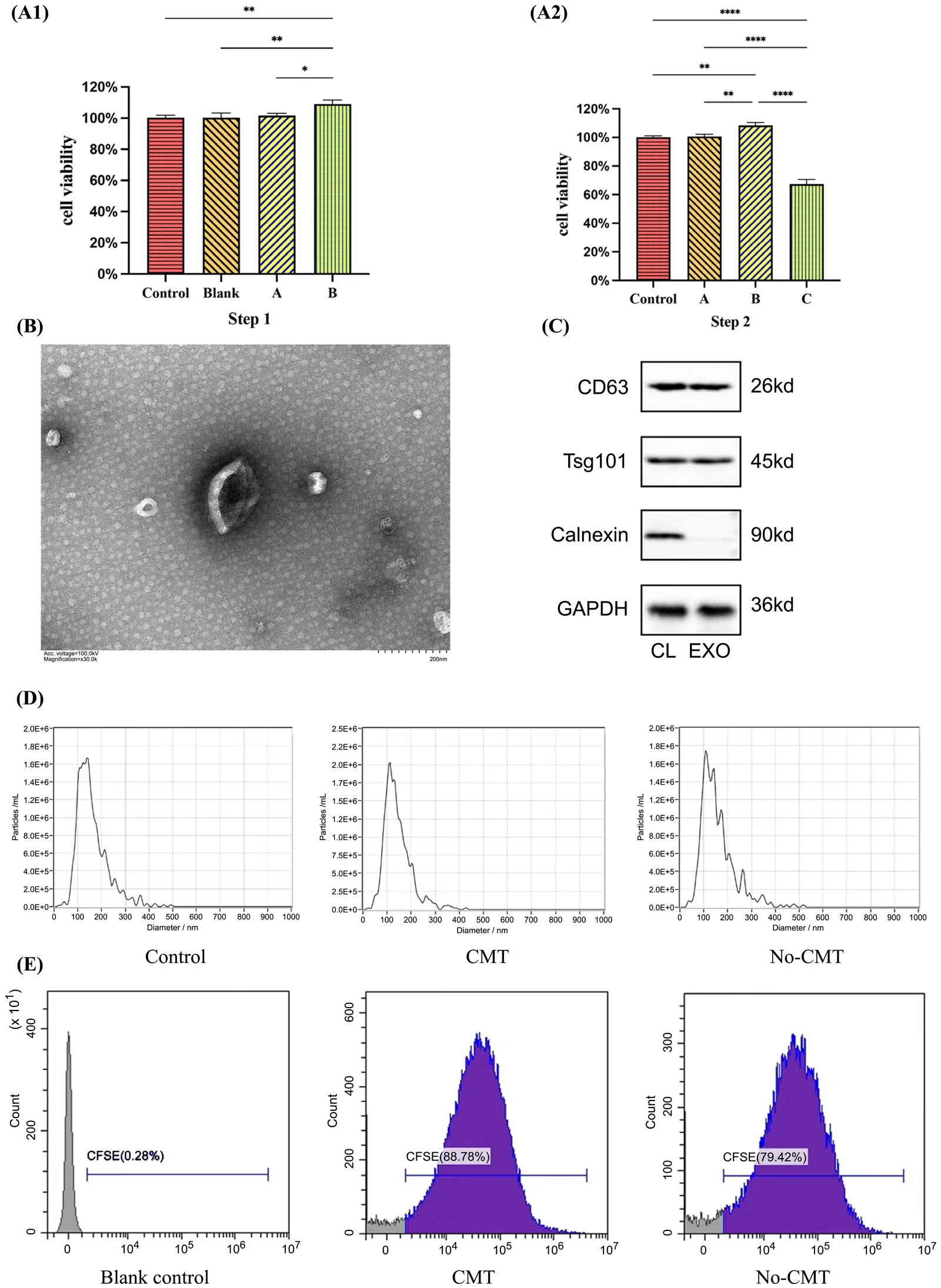
Optimization of CMT parameters and characterization of NPCs-exo. A1-2: Viability assay of NPCs; B: Transmission electron microscopy images of NPCs-exo (scale bar = 200 nm); C: Western blot analysis of NPCs-exo; D: Nanoparticle tracking analysis of NPCs-exo; E: Concentration measurement of NPCs-exo. (∗*p* < 0.05, ∗∗*p* < 0.01, and ∗∗∗∗*p* < 0.0001).

Step 1 CCK-8 analysis revealed comparable NPCs’ viability between Blank (standard culture plates) and Control (Flexcell system) groups, confirming the tension system's non-cytotoxic nature. The B-1 group (10% strain, 1 Hz, 24 hours cyclic tension) demonstrated significantly higher viability than Control, Blank, and A-1 groups (*p*<0.05), establishing these parameters as the screening criteria for Step 2 (Fig. **1A1**).

In Step 2 testing, the B-2 group (maintaining 10%/ 1 Hz/ 24 hours parameters) showed enhanced viability versus Control (*p*<0.05), while A-2 exhibited no significant changes, and Group C displayed markedly reduced viability. This confirmed B-2 parameters as the optimal CMT conditions for subsequent NPCs activation studies (Fig. **1A2**).

TEM revealed that NPCs-exo were circular or elliptical in shape with distinct vesicular membranes. Western blot analysis confirmed the expression of exosome marker proteins CD63 and Tsg101, while the negative marker GAPDH was absent, validating the characteristic membrane protein profile of the extracted NPCs-exo. NTA showed that NPCs-exo had an average diameter of 110.1-134.3 nm, consistent with the typical size range of exosomes (Fig. **1B-D**).

Flow cytometry analysis using CFSE staining was employed to detect NPCs-exo. CFSE passively diffu-ses into cells, with CFSE-positive particles representing intact exosomes. The results demonstrated that both the CMT and No-CMT groups contained abundant exosomes compared to the Blank control group (no dye or test substance added). Moreover, the CMT group exhibited a higher concentration of NPCs-exo than the No-CMT group (Fig. **1-E**).

### 3.2 NPCs Uptake of NPCs-exo Enhances Viability and Reduces Apoptosis

**Fig. (2):**
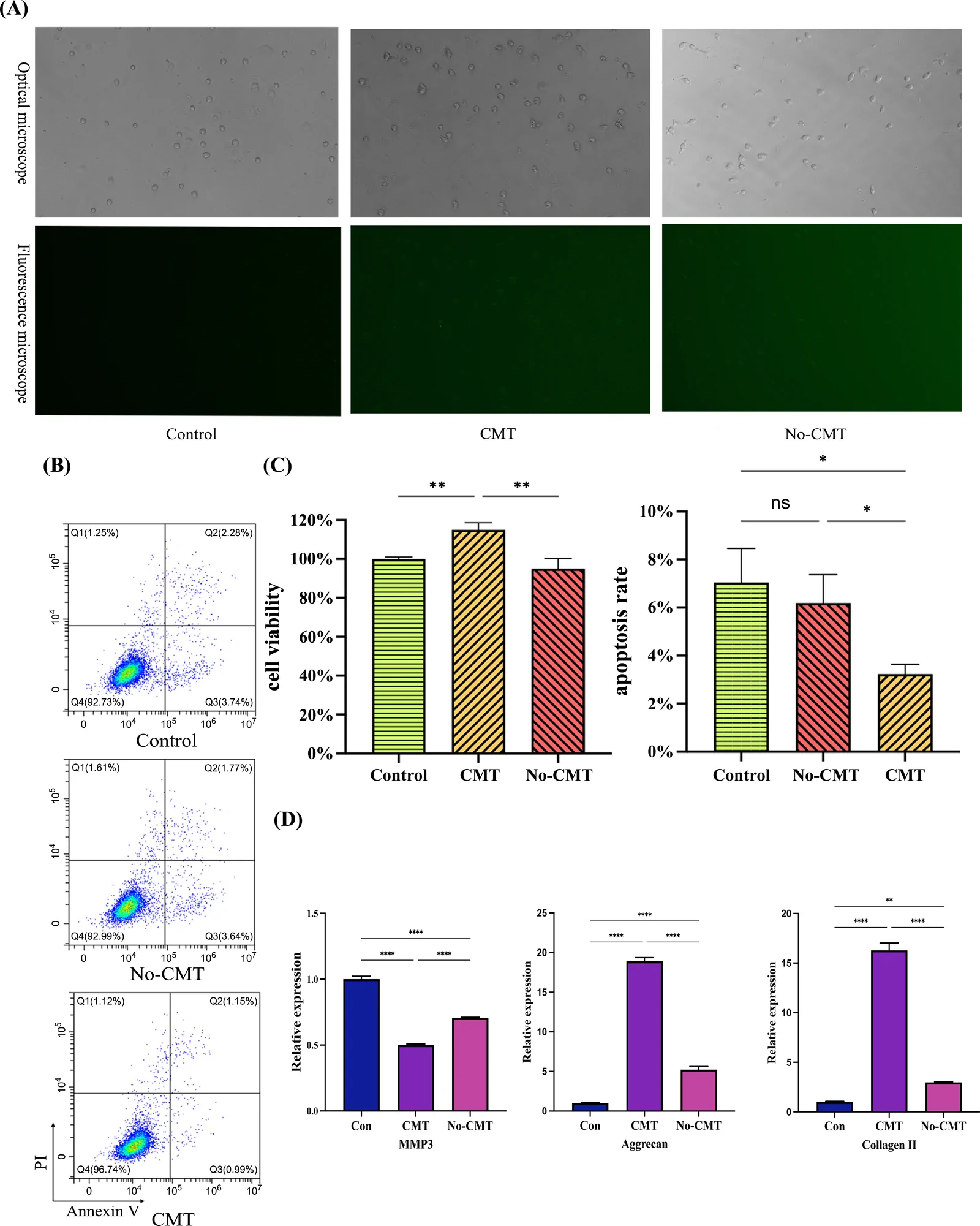
Analysis of outcomes following the co-culture of NPCs-exo and NPCs. A: Fluorescence microscopy images of NPCs co-cultured with NPCs-exo; B: Flow cytometry analysis of NPCs co-cultured with NPCs-exo; C: Assessment of NPC viability and apoptosis; D: qPCR analysis of MMP3, Aggrecan, and Collagen II expression in NPCs. (∗*p* < 0.05 and ∗∗*p* < 0.01).

Following a 24-hour co-culture period involving PKH67-labeled NPCs-exo and NPCs, confocal laser scanning microscopy analysis revealed an absence of green punctate fluorescence in the Control group. In contrast, both experimental groups displayed green punctate fluorescence, confirming the successful uptake of NPCs-exo by NPCs (Fig. **2A**). CCK-8 absorbance assays assessing NPCs proliferation indicated no statistically significant difference between the No-CMT group and the Control group (*P* > 0.05). Conversely, the CMT group exhibited a marked increase in proliferative activity relative to the Control group (*p* < 0.01), suggesting that NPCs-exo secreted under CMT conditions significantly enhance NPCs’ viability (Fig. **2B**).

Flow cytometry analysis was conducted to assess apoptosis in NPCs. The results from the Annexin V-PI staining assay indicated no statistically significant difference in apoptosis rates between the No-CMT group and the Control group (*p* > 0.05). In contrast, the CMT group demonstrated a significantly reduced apoptosis rate compared to the Control group (*p* < 0.05), suggesting that NPCs-exo secreted under CMT conditions may inhibit apoptosis in NPCs (Fig. **2C**). Furthermore, relative mRNA expression analysis revealed that both the CMT and No-CMT groups exhibited significantly elevated levels of Aggrecan and Collagen II mRNA compared to the Control group (*p* < 0.05) (Fig. **2D**). (For qPCR curve, see Supplementary Fig. **1**).

### 3.3 Regulation of the Wnt/β-catenin Pathway via miR-8485 Targeting GSK-3β

**Fig. (3):**
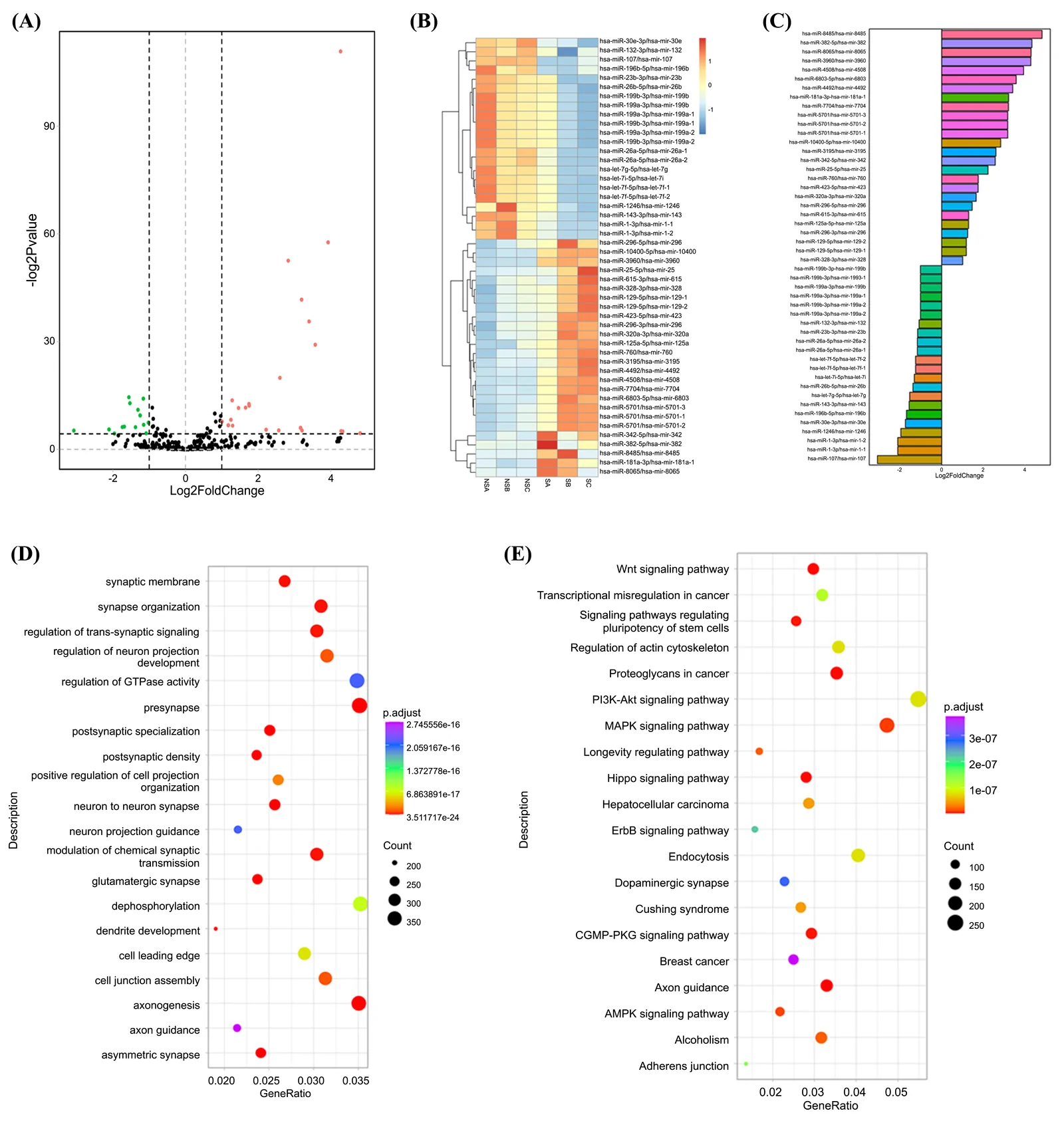
MiRNA high-throughput sequencing outcomes and differential expression selection and analysis. A-B: Volcano plot and heatmap of high-throughput sequencing of miRNAs in NPCs-exo; C: The top 40 miRNAs with the most significant expression changes in NPCs-exo based on high-throughput sequencing; D-E: GO and KEGG enrichment results of differentially expressed miRNAs between the two groups of NPCs-exo.

High-throughput sequencing was utilized to examine miRNA expression profiles in NPCs-exo. By applying differential expression criteria of |log2 (Foldchange)| > 1 and *p* < 0.05, a total of 48 differen-tially expressed miRNAs were identified, comprising 26 upregulated and 22 downregulated miRNAs. Subsequent target gene prediction was conducted for these differentially expressed miRNAs. Enrichment analysis using the KEGG database identified the top 20 pathways, prominently featuring the Wnt signaling pathway, PI3K-Akt signaling pathway, MAPK signaling pathway, and ErbB signaling pathway. Additionally, GO database enrichment analysis underscored functions associated with neuronal and synaptic growth and regulation, trans-synaptic signaling, and mediation and regulation of the cellular environment and movement. To further refine the selection of differentially expressed miRNAs, more stringent criteria were applied: |log2(Foldchange)| > 1 and *p* < 0.05 for the top 10 upregulated miRNAs. In descending order of log2(Foldchange) values, these were: miR-8485, miR-382-5p, miR-8065, miR-3960, miR-4508, miR-6803-5p, miR-4492, miR-181a-3p, miR-7704, and miR-5701. Consequently, miR-8485, which showed the highest upregulation, was selected for this study (Fig. **3A-E**).

**Fig. (4):**
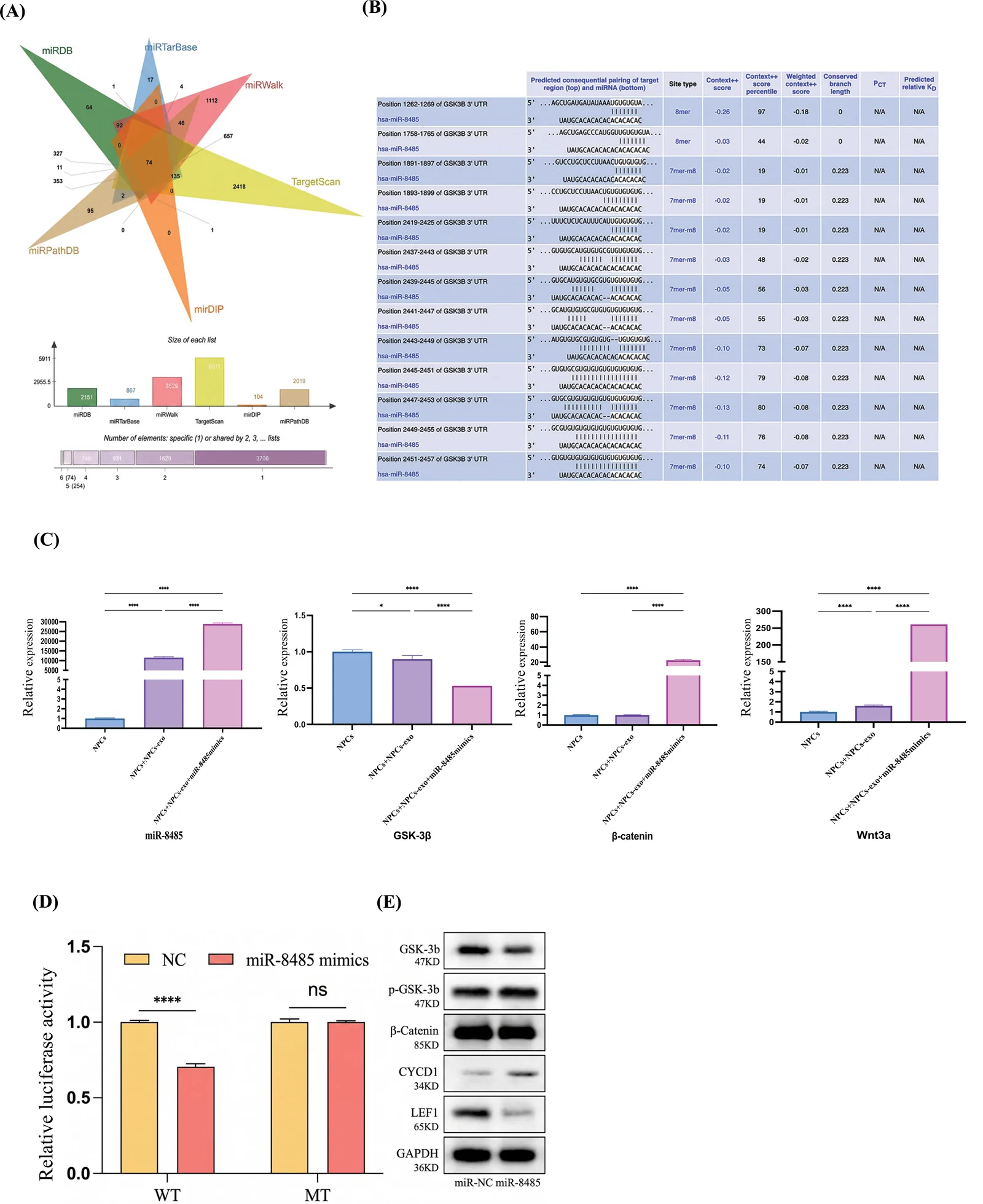
Analysis of miR-8485 target genes, validation of targeting, and staining in animal experiments. A: Venn diagram illustrating miR-8485 target gene predictions based on six databases identified 74 overlapping genes, including GSK-3β; B: Bioinformatics prediction of the miR-8485 binding site on GSK-3β; C: qPCR analysis of miR-8485, GSK-3β, wnt3a, and β-catenin expression; D: Dual-luciferase assay showing the targeting relationship between miR-8485 and GSK-3β, wt = wild-type; MT = mutant.; E: Western blot analysis of key factors in the Wnt/β-catenin pathway following miR-8485 overexpression transfection; (∗*p* < 0.05 and ∗∗∗∗*p* < 0.0001).

Through the use of six target gene prediction databases—TargetScan, miRDB, miRbase, miRTarBase, miRWalk, and miRPathDB—75 inter-secting target genes for miR-8485 were identified, including GSK-3β. (Supplementary 2). The binding sites between GSK-3β and miR-8485 were also predicted (Fig. **4A-B**). By transfecting miR-8485 mimics, overexpression of miR-8485 was achieved. qPCR was employed to measure the expression levels of miR-8485, GSK-3β, wnt3a, and β-catenin in NPCs, NPCs+NPCs-exo, and NPCs+NPCs-exo+miR-8485 mimics groups. This confirmed that miR-8485 can be transported into NPCs through NPCs-exo and can reduce GSK-3β expression while altering the expres-sion of key genes in the Wnt/β-catenin pathway (Fig. **4C**).

A dual-luciferase reporter assay confirmed the targeting relationship between miR-8485 mimics and GSK-3β. Further validation through qPCR and Western blot experiments demonstrated that miR-8485 targets GSK-3β to regulate the Wnt/β-catenin pathway (Fig. **4D-E**).

### 3.4 Evaluation of Tissue Morphology and ECM Indicators in Animal Models

**Fig. (5):**
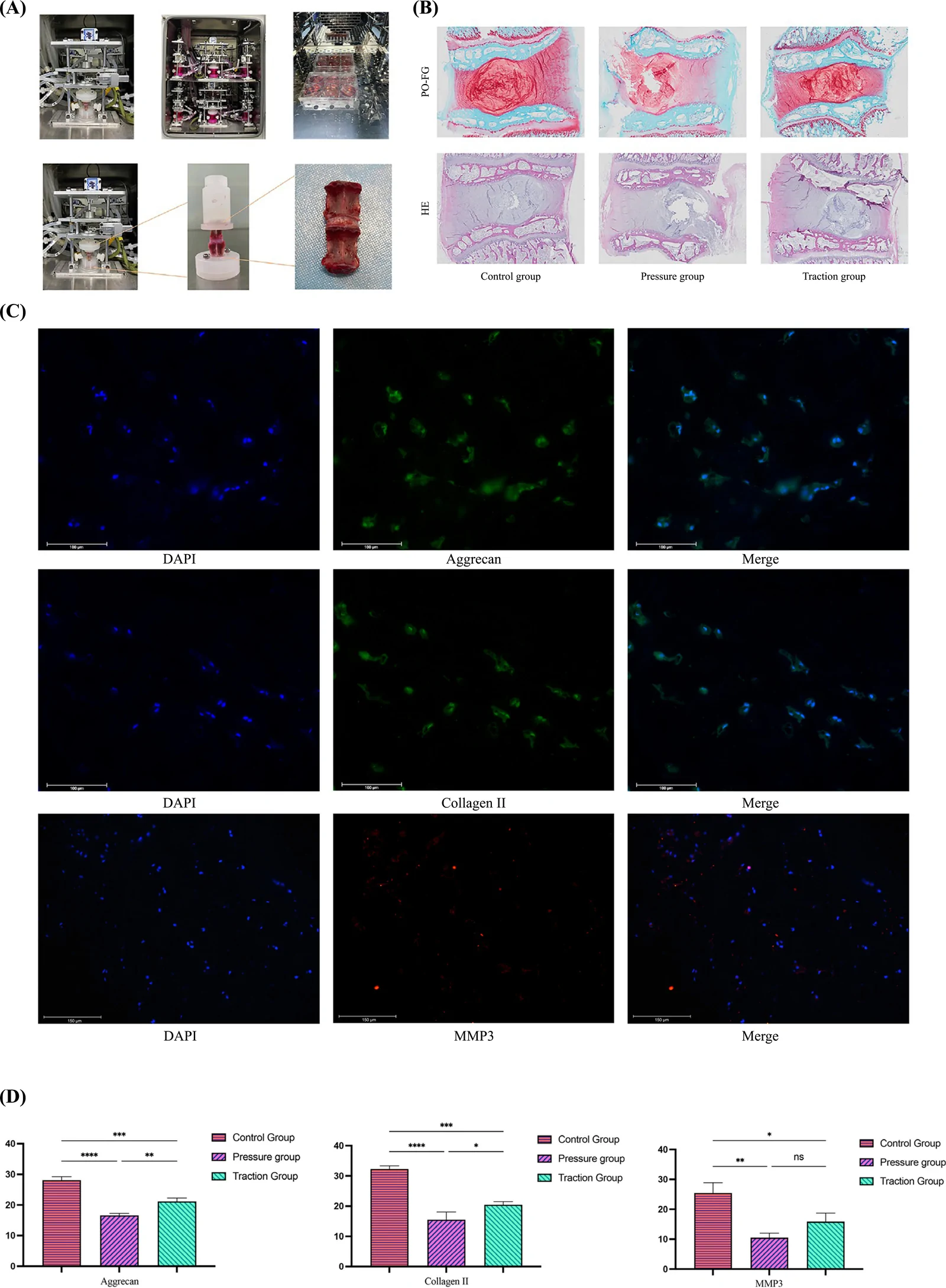
Immunofluorescent staining of IVD sections from animal experiments. A: In vitro loading system and samples of rabbit spinal motion segments; B: HE and PO-FG staining of rabbit spinal motion segments after in vitro loading. C: Immunofluorescence staining of MMP3, Aggrecan, and Collagen II in the isolated spinal motion segments of rabbits after loading; D: Quantitative analysis of immunofluorescence staining results. (∗*p* < 0.05, ∗∗*p* < 0.01, ∗∗∗*p* < 0.001, and ∗∗∗∗*p* < 0.0001).

Safranin O-Fast Green staining revealed that the control group exhibited intact morphology of the NP and CEP, with orderly cell arrangement and abundant chondrocytes. The pressure group showed cartilage endplate deformation under abnormal pressure, while the traction group demonstrated notably improved tissue deformation, albeit with some degree of cartilage endplate deformation and chondrocyte loss still present (Fig. **5A**-**B**).

H&E staining results indicated that the control group maintained an ordered overall structure of the IVD tissue, with abundant NPCs visible in the NP, and an orderly AF structure encasing the NP without rupture. The pressure group, under prolonged abnormal pres-sure, exhibited AF rupture, NP morphological abnormalities with extrusion, and significant NPCs loss. The traction group exhibited better morphology compared to the pressure group, with relatively ordered AF and NP structures and higher NPCs content, although some NPCs loss was still observed.

Regarding ECM-related indicators Collagen II, Aggrecan, and MMP3, both pressure and traction groups showed significant differences compared to the control group (p < 0.05). The traction group exhibited significant differences in Collagen II and Aggrecan expression compared to the pressure group (p < 0.05) (Fig. **5C-D**).

## 4 DISCUSSION

In this study, CMT was employed to simulate traction forces, and its effects were investigated on NPC-exo secretion at the cellular level. Appropriate mechanical stimulation has been shown to regulate cell growth and differentiation [30]. CMT is a common method of applying mechanical stimulation in cellular studies. The impact of CMT on cells is mainly demonstrated through its regulation of cell proliferation and differentiation [31, 32]. Considering the importance of CMT in IVDD research, the widely accepted Flexcell tension system, a computer-controlled bioreactor, was employed. These bioreactors apply static and cyclic strain to different cell types cultured as 2D monolayers or embedded in 3D hydrogel models, enabling the evaluation of cellular responses to mechanical strain [33, 34]. This device has been widely used in published research and is applicable to almost all cell types, including chondrocytes, stem cells, and neuronal cells [35-39]. As a result, it ensures stable and sustained CMT loading during NPC culture.

Additionally, CMT can alter the expression of various intracellular miRNAs and participate in the progression of multiple human diseases by regulating the relationship between miRNAs and their target cells [40]. In IVD cell research, a single-cell transcriptomic analysis revealed biomechanical loading induces an imbalance that leads to ossification during NPCs degeneration [41]. CMT has been shown to regulate matrix metabolism, autophagy, and cytoskeletal arrangement in cartilage endplate cells through MAPK and Wnt/β-catenin pathways [42]. Prolonged CMT can induce calcification of cartilage endplate cells and downregulate ankh gene expression [43], while short-term CMT can increase ankh gene expression in these cells via TGF-β1 and p38 pathways [44]. However, studies applying CMT to NPCs are relatively scarce. Feng *et al.* [45] demonstrated that under conditions of 20% strain at 1 Hz frequency, NPCs exhibited significant premature senescence following activation of the p53-p21-Rb pathway. CMT at 20% strain can induce endoplasmic reticulum stress and apoptosis in rat annulus fibrosus cells [46]. In summary, CMT has been shown to participate in the regulation of complex biological processes and signaling pathways in IVD.

We examined changes in NPCs’ viability, apoptosis, ECM production, and Wnt/β-catenin pathway-related factors through co-culture of NPCs with NPC-exos under CMT conditions. Previous studies have demonstrated that 20% strain can significantly induce apoptosis in NPCs [20, 45]. Therefore, to maximize NPC viability, we screened CMT parameters to avoid excessive apoptosis that might reduce NPC-exo secretion. Results from NPC viability assays following CMT application revealed that parameters of 10% strain, 1 Hz frequency, for 24 hours effectively promoted NPC viability, while higher CMT parameters significantly reduced it. Consequently, we selected these parameters for CMT loading to efficiently isolate NPC-exos. Previous research has similarly shown that mechanical stimulation within certain limits can promote cell proliferation. Wahlsten *et al.* [47] induced rapid fibroblast proliferation through mechanical stretching, while Yang *et al.* [48] found that CMT enhanced human periodontal ligament cell proliferation and differentiation through YAP activation.

By co-culturing NPC-exos with NPCs, we demonstrated that NPCs can efficiently take up NPC-exos. First, exosomal transmembrane proteins can directly interact with signaling receptors on target cells [49]. Second, exosomes can fuse with the recipient cell membrane and deliver their contents into the cytoplasm [50]. Third, exosomes can be internalized by recipient cells through endocytosis, potentially traversing the recipient cell and being released to adjacent cells, or being degraded within the recipient cell [51]. Building on this foundation, we examined NPCs after NPC-exo uptake. The results demonstrated that CMT-induced NPC-exosomes effectively promoted NPC proliferation and inhibited apoptosis. Previous research [52] has shown that exosomes can regulate NPC viability, apoptosis, and ECM synthesis by influencing the Wnt/β-catenin pathway. Therefore, we hypothesized that NPC-exosomes might also affect NPCs by modulating the Wnt/β-catenin pathway. GSK-3β can phosphorylate β-catenin, leading to its degradation and inactivation, thus reducing Wnt/β-catenin pathway activity. GSK-3β can phosphorylate β-catenin, leading to its degradation and inactivation, thus reducing Wnt/β-catenin pathway activity [53-55].

The Wnt/β-catenin pathway plays a crucial role in stem cell renewal, cell proliferation, and differentiation during embryonic development and adult tissue homeostasis [56-58]. In the context of IVD, Wnt signaling plays a crucial role in regulating disc cell function and maintaining matrix homeostasis. Its dysregulation has been linked to heightened catabolic activity, leading to increased ECM degradation and inflammation. In the canonical Wnt/β-catenin pathway, activated β-catenin accumulates in the cytoplasm and translocates to the nucleus, initiating downstream target gene transcription [59]. The Wnt/β-catenin signaling pathway is interconnected with several other known pathways associated with IVD, forming a complex interaction network that influences the pathogenesis of IDD. It interacts with pathways such as Transforming Growth Factor-β (TGF-β) and Bone Morphogenetic Protein (BMP), thereby regulating ECM synthesis and cellular differentiation. Additionally, its crosstalk with the NF-κB signaling pathway modulates cellular senescence and ECM metabolism in disc degeneration [60]. This interaction can regulate the expression of enzymes, such as MMPs, and the degradation of the extracellular matrix, thereby influencing intervertebral disc health, which is consistent with the findings of this study. Moreover, numerous studies have demonstrated that the Wnt/β-catenin signaling pathway can be regulated by exosome-derived miRNAs. MiR-31 carried by bone marrow mesenchymal stem cell-derived exosomes [61] inhibits NFAT5 expression, leading to Wnt/β-catenin pathway activation, thereby promoting NPC proliferation and reducing cell apoptosis and ECM degradation. Conversely, miR-532 downregulates Wnt/β-catenin signaling and induces human nucleus pulposus cell apoptosis by targeting Bcl-9 [62].

NPCs have been shown to be one of the internal factors responsible for IVDD, as they can target MMP-13 and prevent extracellular matrix degradation by delivering EXO miR-27a via autophagy [63]. At the same time, due to their degeneration, exosomes from NPC stem cells in degenerated intervertebral discs exacerbate annulus fibrosus cell degradation via Let-7b-5p [64]. This study provides evidence of the influence of NPCs-exo on IVDD under mechanical changes. MiR-8485 is a miRNA that has not been extensively studied, but existing research indicates its role in arthritis and cartilage degeneration. MiR-8485 alleviates IL-1β-induced chondrocyte inflammation by suppressing CRLF1 expression [65]. It can regulate the Wnt/β-catenin signalling pathways to facilitate chondrogenesis [66]. Our research also corroborates its role in regulating the Wnt/β-catenin pathways, which are implicated in bone and joint degenerative diseases. This study marks the first instance of its application in conjunction with EXO, demonstrating significant potential for future research into related diseases. Moreover, compared to other miRNAs, miR-8485 exhibits a distinct advantage in its sensitivity to the biomechanics of the IVD. It shows significantly differential expression in EXO under mechanical stress [67]. Given that mechanical factors are an inevitable contributor to the pathogenesis of IVDD and represent a primary physiological function of the disc [68, 69], these characteristic underscores the unique role of miR-8485 in IVDD research. Studies have reported that EXOs secreted by degenerated NPCs can induce macrophage M1 polarization, thereby promoting IVDD [70]. The role of miR-8485 in this process may be a promising area for further investigation.

SU loading is a commonly used technique in *ex vivo* IVD degeneration models [9, 71]. Previous studies have demonstrated that under sustained compressive loading of the FSU, IVDs initially undergo morphological changes. With prolonged loading, the annulus fibrosus gradually stretches and deforms, the nucleus pulposus shifts from the central region of the IVD, and the expression of collagen type II and aggrecan in IVD tissues decreases [26, 72]. Additionally, vascular budding and VEGFA expression in the cartilage endplate progressively decline [29].

Conservative treatment is the preferred approach for IVDD-related diseases, encompassing methods such as pharmacotherapy, rehabilitation exercises, traction, physiotherapy, and manual manipulation [73-75]. Traction is one of the most used and effective treatments for certain spinal disorders [76-79], with a favorable safety profile [80]. However, the underlying mechanisms of traction therapy remain inadequately elucidated. In this study, we employed an *ex vivo* model combined with cellular experiments to demonstrate the potential mechanism by which traction contributes to the attenuation of IVDD. Currently, much research has been carried out on the mechanical pathogenic factors of IVDD, but studies on mechanical treatment factors are limited. We believe that more attention should be given to the basic research on traction therapy for IVDD. To this end, we have developed an *ex vivo* experimental platform featuring a fully sterile liquid circuit and computer-controlled mechanical and fluidic modulation. This system enables precise regulation of both mechanical and culture environments, allowing for *ex vivo* spinal tissue cultivation with scientifically managed conditions. Furthermore, it facilitates three-dimensional mechanical loading, including tensile, compressive, and rotational forces [29, 81].

## STUDY LIMITATIONS

In this study, an *ex vivo* rabbit model was utilized, and extensive experiments were conducted to optimize the device for better simulation of the human spinal biomechanical environment. Additionally, we aimed to ensure that the device could withstand both compressive and tensile forces. However, inherent differences between human and rabbit spinal microenvironments, such as variations in nutrient diffusion and vertical loading patterns, remain unavoidable. Furthermore, the research was confined to axial stress, whereas the IVD in humans experiences multi-directional forces. These limitations necessitate further in-depth investigations in future studies.

## CONCLUSION

CMT enhances the secretion of NPCs-exo, which are internalized by NPCs, resulting in elevated NPCs activity and reduced apoptosis. Mechanistically, these exosomes deliver miR-8485 to target and modulate GSK-3β, thereby activating the Wnt/β-catenin pathway. This cascade promotes NPCs viability and extracellular matrix synthesis while suppressing apoptosis, ultimately attenuating IVDD progression. Animal experiments validated the *in vitro* findings, with immunofluorescence staining demonstrating that mechanical traction robustly enhances extracellular matrix expression in the intervertebral disc and ameliorates stress-induced morphological degeneration.

## Figures and Tables

**Table 1:** Experimental grouping for CMT parameter screening.

**Group**	**Traction Rate**	**Frequency**	**Duration**
Step 1	-	-	-
Control	0	0	24 hours
Blank	0	0	24 hours
A	5%	1Hz	24 hours
B	10%	1Hz	24 hours
Step 2	-	-	-
Control	0	0	24 hours
A	X	0.5Hz	24 hours
B	X	1Hz	24 hours
C	X	5Hz	24 hours

**Table 2:** Primer sequences.

**Item**	**Primer sequences**
Collagen II	F: TGGACGCCATGAAGGTTTTCT;
	R: TGGGAGCCAGATTGTCATCTC
Aggrecan	F: GCCCAAGACTACCAGTGGAT
	R: GCGTTTGTAGGTGGTGGCTG
MMP3	F: CTGGACTCCGACACTCTGGA;
	R: CAGGAAAGGTTCTGAAGTGACC
GSK-3β	F: GGCAGCATGAAAGTTAGCAGA;
	R: GGCGACCAGTTCTCCTGAATC
Wnt3a	F: TAGGAAGAGAGGTCCAGCCC;
	R: CTCCAGGAAAGCGGACCATT
β-catenin	F: AAAGCGGCTGTTAGTCACTGG;
	R: CGAGTCATTGCATACTGTCCAT
GAPDH	F: TCATTGACCTCAACTACATGG;
miR-8485	R: TCGCTCCTGGAAGATGGTG F: TCGGCAGGCACACACACACACAC; R: CTCAACTGGTGTCGTGGAGT

## Data Availability

All the data and supportive information are provided within the article.

## LIST OF ABBREVIATIONS

IVDD: Intervertebral disc degeneration
NP: Nucleus pulposus
NPCs: Nucleus pulposus cells
NPCs-exo: NPCs-derived exosomes
CEP: Cartilaginous endplate
CEPCs: Cartilaginous endplate cells

## RESEARCH INVOLVING ANIMALS

All experimental procedures involving animals were conducted in accordance with the standards outlined in the 8th edition of the Guide for the Care and Use of Laboratory Animals. This study adheres to internationally accepted standards for animal research, following the 3Rs principle. The ARRIVE guidelines were employed for reporting experiments involving live animals, promoting ethical research practices.
